# Chitosan-functionalized mesoporous silica nanoparticles co-loaded with chrysin and quercetin: a potent strategy against lung cancer cells

**DOI:** 10.1038/s41598-025-29511-3

**Published:** 2026-01-05

**Authors:** Chou-Yi Hsu, Ammar Yasir Ahmed, Nahed S. Alharthi, Alanood S. Algarni, Fakhria A. Al Joufi, R. Roopashree, Zafar Aminov, Sumit Kaushal, Firas Sattar Gheni AL-Jabban, Khursheed Muzammil

**Affiliations:** 1 Department of Pharmacy, Chia Nan University of Pharmacy and Science, Tainan, 71710 Taiwan; 2 College of Pharmacy, University of Al Maarif, Al Anbar, 31001 Iraq; 3 Department of Medical Laboratory, College of Applied Medical Sciences in Al-Kharj, Prince Sattam Bin Abdulaziz University, Al-Kharj, 11942 Saudi Arabia; 4 Pharmacology and Toxicology Department College of Pharmacy, Umm Al-Qura University, Makkah, Saudi Arabia; 5 Department of Pharmacology, College of Pharmacy, Jouf University, Aljouf, 72341 Saudi Arabia; 6 Department of Chemistry and Biochemistry, School of Sciences, JAIN (Deemed to be University), Bangalore, Karnataka India; 7 Department of Public Health and Healthcare management, Samarkand State Medical University, Amir Temur Street, Samarkand, Uzbekistan; 8 Centre for Research Impact & Outcome, Chitkara University Institute of Engineering and Technology, Chitkara University, Rajpura, 140401 Punjab India; 9 Anesthesia Techniques Department, College of Health and Medical Techniques, Al-Mustaqbal University, Babylon, 51001 Iraq; 10 Department of Public Health, College of Applied Medical Sciences, King Khalid University, Khamis Mushait Campus, Abha, Kingdom of Saudi Arabia

**Keywords:** Lung cancer, Dual drug-loaded, Mesopurs silica nanoparticle, Quercetin, Chrysin, Apoptosis, Cell cycle, Biochemistry, Biotechnology, Cancer, Drug discovery, Molecular biology

## Abstract

Lung cancer (LC) represents a major and growing challenge in global healthcare, necessitating the exploration of innovative therapeutic strategies. In this context, nanoparticles (NPs) have emerged as promising platforms for enhancing treatment efficacy and improving patient outcomes. The present study investigated the cytotoxic effects of chitosan-functionalized mesoporous silica nanoparticles (MSNs) co-loaded with chrysin (Chr) and quercetin (Qur)—denoted as Chr–Qur@MSNs–Chi—on A549 lung cancer cells. Chr–Qur@MSNs–Chi NPs were synthesized and characterized using dynamic light scattering (DLS), Fourier transform infrared spectroscopy (FTIR), and scanning electron microscopy (SEM). Cell viability and apoptosis-related gene expression were evaluated using the MTT assay and quantitative real-time PCR (qRT-PCR), respectively.The synthesized NPs were spherical, with an average size range of 80–110 nm, and exhibited no detectable impurities. The DLS analysis indicated a particle size of approximately 110 nm and a zeta potential of − 36.5 mV. The MTT assay revealed IC₅₀ values of 1 µM and 2 µM after 24 and 48 h of treatment, respectively. Furthermore, Chr–Qur@MSNs–Chi induced greater cell cycle arrest at the G0/G1 phase compared to the free Chr–Qur combination. Gene expression analysis demonstrated significant upregulation of p53, Bax, and Fas (2.1-, 2.2-, and 2.4-fold, respectively), alongside downregulation of Cyclin D1, pRB, and Bcl-2 (0.6-, 0.8-, and 0.7-fold, respectively), indicating strong apoptotic effects (*P* < 0.001). These findings suggest that Chr–Qur@MSNs–Chi nanoparticles exhibit potent anticancer activity against A549 human lung cancer cells, likely through the induction of apoptosis and modulation of apoptosis-related gene expression pathways.

## Introduction

According to the International Agency for Research on Cancer (GLOBOCAN), lung cancer remains the leading cause of cancer-related mortality worldwide, accounting for approximately 18% of all cancer deaths^1^. Considering its biological characteristics, prognosis, and response to therapy, lung cancr is expansively classified into two main categories: small cell lung cancer (SCLC)^2^ and non-small cell lung cancer (NSCLC). Within this spectrum, NSCLC is the most prevalent subtype^3^.

Various approaches have been employed to manage lung cancer, including surgical intervention, chemotherapy, and radiation therapy^4,5^. Nevertheless, achieving a complete cure with these treatments challenging and often short-lived, especially in advanced stages of the disease^6^. Chemotherapy plays a pivotal role in treatment of NSCLC, however, it faces significant challenges, including the emergence of frug resistance and the severe off-target toxicities. These outcomes not only decrease the efficiency of clinical interventions but also increase the risk of serious unfavorable outcomes, including mortality^7,8^. Therefore, there is an urgent and vital clinical need to develope innovative and improved strategies for the treatment of NSCLC.

A broad variety of natural compounds have been applied for an extended period as remedies for tumors. Among them, Chr and Qur has been confirmed to displays significant anti-cancer effects and are present in a diversity of medicinal plants^9^. Qur displays a broad spectrum of biological and pharmacological activities, including anti-inflammatory, antidiabetic, antioxidative, anticancer properties. Chr (5,7-dihydroxyflavone), a polyphenolic flavone known to act roles in diverse pathways, notably in preventing, delaying, or reversing the development of cancer, is frequently found in fruits, vegetables, and honey^10^. It exhibits anti-inflammatory, antioxidant^11^, immunomodulatory^12^, and anti-cancer attributes^13^. Therefore, the co-administration of Chr with Qur may enhance the therapeutic efficacy of this compund through different mechanisms. Despite their promising health benefits, the limited water solubility, poor absorption, low stability and rapid metabolism of Chr and Qur present substantial challenges to their effective use in medicine, principally in cancer treatment^10^.

The rapid evolution of nanotechnology has greatly simplified the discovery and utilization of nanocarriers for drug delivery^14,15^. These nanocarriers includes both organic types- such as nanoparticles, liposomes and micelles- and inorganic types, including gold NPs, quantum dots, magnetic nanocarriers, carbon-based materials and mesoporous silica nanoparticles (MSNs)^16^.

Among various nanocarriers, MSNs have attracted increasing attention from researchers in the field of cancer therapy, principally for the treatment of lung cancer^17^. MSNs have a sturdy, high–surface-area structure that can trap poorly soluble phytochemicals inside their tiny pores, helping these compounds dissolve and spread more easily in water-based environments. To enhance the drug delivery efficiency and decrease the adverse effects related with numerous anti-tumor medications, several kindes of stimuli-responsive mesoporous silica nanoparticles have been established, capable of discharging therapeutic agents in response to exterior stimuli such as pH, magnetic fields, temperature and light^18^. Newly, pH-responsive delivery systems have emerged as a hopeful approach for tumor therapy because of the capability of NPs to be delivered to acidic tumor environments via the enhanced permeability and retention (EPR) effect. Once internalized, the NPs are usually entangled in acidic endosomes, which have a pH range of 5.5 to 6.0^19^. Thus, this inspires investigators to attention on the development of pH-responsive drug delivery system, which would simplify a faster drug discharge rate at lower pH levels compared to physiological (neutral) pH.

**Fig. 1:**
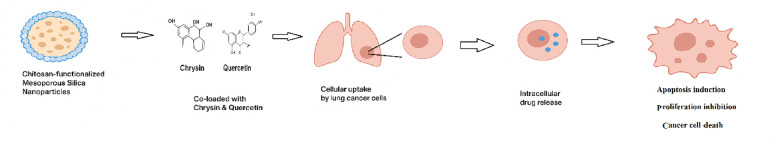
A schematic illustration of this study.

Hence, the current work rationally designed to develop of pH-responsive composite microsphere as carriers fo combination therapy with Qur and Chr, in order to facilitate the delivery of NPs to the tumor cells. A schematic illustration of this work is depicted in (Fig. 1).

## Materials and methods

### Material

Qur, Chr (with a purity exceeding 99%), Chitosan, DAPI (4’,6-diamidino-2-phenylindole), Tetraethylorthosilicate (TEOS), and 3-Aminopropyltriethoxysilane (APTES) were sourced from Sigma-Aldrich (St. Louis, MO, USA). Dimethyl sulfoxide (DMSO) was acquired from Merck (Darmstadt, Germany). Cell culture materials, including 3-(4,5-dimethylthiazol-2-yl)-2,5-diphenyltetrazolium bromide (MTT), Roswell Park Memorial Institute (RPMI)-1640 medium, fetal bovine serum (FBS), trypsin–EDTA, penicillin, and streptomycin, were obtained from Gibco (AG, Basel, Switzerland). Annexin V/PI was acquired from eBioscience (San Diego, CA, USA). All other chemicals were supplied by Merck and subjected standard purification procedures.

### Cell lines and cell culture

The human lung cancer cell line A549 was bought from the Cell Bank at the Pasteur Institute of Iran. RPMI-1640 medium was used for culturing the A549 cells. Prior to utilization, the media were enriched with 1% penicillin-streptomycin and 10% FBS. The cells were cultured in 37 °C, 5% CO_2_, and 95% humidity, and the media were replaced every two days. For most experiments, cells that achieved confluence levels between 80% and 90% were subjected to subsequent analysis. Detailed protocols for the application of other reagents are provided in separate sections.

### MSNs synthesis

The preparation of MSNs was done using the sol-gel technique, as a described previously. Fisrt, 1 gram of CTAB was dissolved into 480 ml of DI water under stirring, and the temperature of the solution was retained at 80 °C. Next, a solution of hydroxide (2 M, 3 ml) was introduced to the CTAB mixture. Then, the mixture was stirred steadily for 20 min before 5 ml TEOS was added slowly in a dropwise manner to the CTAB solution, permitting the reaction to remain for another 2 h. Afterward, solid crude product was acquired after the mixture was retained for at least 12 h. The obtain product was rinsed thoroughly several times with DI water and ethanol, and then dried in a vacuum at 50 °C overnight to produce the MSNs.

### Fabrication and optimization of Chr-Qur @MSNs-Chi NPs

MSNs were optimized for uniform size (~ 100–120 nm), high surface area (~ 800 m²/g), and a well-defined mesoporous structure to enhance drug loading. Synthesis parameters such as surfactant concentration, silica precursor ratio, pH, and temperature were carefully adjusted for stability and efficiency. Chitosan coating was applied to improve biocompatibility and enable sustained release of Qur and Chr. Chr-Qur @MSNs-Chi NPs were prepared following previously reported protocols^18^. Briefly, MSNs (500 mg), Chr (20 mg), and Qur (20 mg) were ultrasonically dispersed in 10 mL of acetone/ethanol (30:70, v/v) and stirred at 37 °C overnight. The mixture was centrifuged at 5000 rpm for 30 min at 5 °C, and the pellet was collected and vacuum dried at 55 °C for 24 h to remove residual solvent. The 1:1 ratio of Chr to Qur was selected based on preliminary cytotoxicity assays in A549 cells, providing synergistic anticancer effects while maintaining measurable dose-dependent responses, resulting in a formulation with efficient drug loading and enhanced therapeutic efficacy.

### Physicochemical characterization of NPs

#### FTIR analysis

Fourier Transformed Infrared (FTIR) spectroscopy was performed using a spectrometer (Shimadzu 8400s, Kyoto, Japan) in the range of 4000 to 400 cm⁻¹ to evaluate the characteristic chemical bonds and molecular interactions within the Chr-Qur@MSNs-Chi NPs. The analysis was conducted using a KBr pellet.

#### Measurements of particle size distribution, polydispersity index (PDI), and zeta potential

Dynamic light scattering (DLS) Zetasizer Nano ZS (Malvern Instruments Ltd., Malvern, UK) was employed to measure average hydrodynamic size (nm), polydispersity index (PDI) and zeta potential (ζ-potential, mv) of the of synthesized Chr-Qur@MSNs-Chi NPs. The results were presented as means ± standard deviation, based on three independent measurements.

#### X-ray diffraction

The crystallographic structure of the prepared Chr-Qur @MSNs-Chi NPs was evaluated through X-ray diffraction (XRD) analysis using an XRD-6000 instrument from Shimadzu, Japan. This measurements were utilized X-ray radiation with a wavelength of 1.54056 Å and covered a scanning rate of 1 s (using Cu-Kα radiation, λ = 1.5406 Å).

#### Nitrogen adsorption–desorption measurements

Nitrogen adsorption–desorption measurements were done at the temperature of liquid nitrogen (77 K). The sample’s surface area was determined using the Brunauer–Emmett–Teller (BET) method, while the pore size distribution was analyzed with the BJH approach.

### NPs morphology

#### Transmission electron microscopy

The structure and morphology of Chr-Qur@MSNs-Chi NPs were examined using a JEOL 3010 high-resolution transmission electron microscope (HRTEM, Tokyo, Japan). The dissolved NPs in deionized water was drop-casted onto carbon copper grids, air-dried, and then evaluated at an accelerating voltage of 200 kV.

#### FE-SEM analysis

The surface properties, structure, morphology and particle size of the prepearded Chr-Qur@MSNs-Chi NPs were studied using a field emission scanning electron microscopy (FE-SEM) (MIRA3 TESCAN). All samples were sputter coated with a thin layer of gold.

### Drug loading

The encapsulation efficiency (EE) of the anticancer compound, Qur and Chr, whitin the MSNs-Chi NPs was determined using an indirect technique. Firstly, a standard calibration curves were constructed by preparing a series of Qur and Chr solutions with known concentrations. The absorbance of these standard mixtures was calculated using UV–visible spectrophotometry. After prepareation of Chr-Qur@MSNs-Chi NPs, the dispersion was centrifuged at 12,500 rpm, 30 min. The amount of Qur and Chr in the supernatant were measured to calculate the encapsulation efficiency using Eqs. 1 and 2 .

Qur and Chr loading efficiency was assessed according to the Eqs. (1, 2):

1 $$\:DL\:\left(\%\right)=\:\frac{Mass\:of\:drugs\:in\:NPs}{Total\:NPs\:mass}\:\times\:100$$

2 $$\:EE\:\left(\%\right)=\:\frac{Mass\:of\:drugs\:in\:NPs}{Total\:mass\:drug}\:\times\:100$$

### In vitro drug release

Phosphate buffered saline (PBS, pH 7.4) and acetic buffer solutions (ABS, pH 5) were used as the drug release media to simulate normal blood/tissues and tumor environments. To evaluate the cumulative release profile of Chr and Qur from MSNs-Chi NPs, a dialysis technique was applied. Briefly, 20–30 mg of the prepared NPs were dissolved in 5 ml of PBS (pH 5 and 7.4) to mimic normal blood/tissues and cancer environments and subsequently introduced into dialysis membrane tubing with a molecular weight cutoff of 3000 Da. Following this, dialysis bags were then submerged in 15 ml of PBS and agitated at 150 rpm at 37 °C to carry out an in vitro drug discharge studies. The levels of released Qur and Chr in the release medium were measured by employing a calibration curves. At predetermined time intervals, the absorbance was conducted using a UV-Vis spectrophotometer (Varian-Cary100, Australia) at wavelengths corresponding to the peak absorbance of Qur (258 nm) and Chr (234 nm). The cumulative proportions of released Qur and Chr are calculated over time.

### Hemolysis assay test

The cytotoxic effect of NPs was measured through a hemolysis test. In the current analyze, PBS and Triton X-100 were served as negative control and positive control, respectively. The blood sample (10 ml) was collected, and 5 ml was moved into tubes containing 7 ml 0.2 M D-PBS.The resulting mixture was centrifuged at 3800 rpm for 10 min, and the resulting pellet, containing red blood cells (RBCs), was consequently rinsed with D-PBS. The RBCs were then diluted with 30 ml of D-PBS. The samples were incubated at room temperature for 4 h, followed by vortexing and centrifugation at 3800 rpm for 4 min. The supernatant was gathered, and the optical density (OD) was measured by absorbance measurement at 540 nm. Hemolysis was quantified by comparing the OD values to the standard controls.

### Cytotoxicity assay

The assessment of cytotoxicity was conducted using the MTT test, which measures the conversion of a water-soluble tetrazolium salt into formazan by the mitochondrial dehydrogenases of the viable cells. A549 cells were cultured at a density of 2 × 10^4^ cells per well in RPMI 1640 medium supplemented with 10% FBS and 1% penicillin/streptomycin, and incubated overnight at 37 °C. Then, the cells were exposed for 24 and 48 h to various concentrations of the blank MSNs, MSNs, free Chr, free Qur, Qur–Chr mixture, Qur NPs, Chr NPs, and Chr-Qur @MSNs-Chi NPs. After the determined time of exposure, the culture medium was substituted with 200 µM MTT solution, and cells were incubated for an additional 4 h. To dissolve the formazan crystals and acquire a liquid form of formazan, 200 µL of DMSO was added, followed by an additional 20 min incubation. Aorbance was measured at 570 nm using an EL 800 microplate absorbance reader (Bio Tek Instruments, Winooski, VT), with a baseline comparison made at 630 nm. The experiments were conducted a total of three times.

### Examination of nuclear alterations

The nuclear morphology of A549 cells was examined using fluorescence microscopy. Cells were cultured at a density of 2 × 10^5^ cells/mL in a 6-well plate and treated with IC_50_ concentrations of free Chr, free Qur, Qur -Chr, Qur NPs, Chr NPs, and Chr- Qur @MSNs-Chi NPs for 48 h. Following treatment, the cells were washed with PBS and then fixed in 2.5% glutaraldehyde for 20 min. Subsequently, the cells were permeabilized in 0.1% Triton X-100 for 15 min and then stained with DAPI (1 µg/mL) for 20 min at room temperature in the dark. After three whashes with PBS, the the nuclear morphology of the A549 cells was assessed using fluorescence microscopy (Olympus, Japan).

### Analysis of cell cycle arrest

The progression of the cell cycle was evaluated using FACS analysis following DNA labeling with propidium iodide (PI). A549 cells were cultured for 24 h and then treated with IC_50_ concentrations of free Chr, free Qur, Qur -Chr, Qur NPs, Chr NPs, and Chr- Qur @MSNs-Chi NPs for 48 h. After being incubated for 48 h, the cells were washed twice with PBS, trypsinized, collected, and subsequently submerged in 70% ethanol at 4 °C for for 30–60 min for fixation. After the fixation process, 10 µL ribonuclease A was added to tubes and then allowed to incubate at 37 °C for 45 min in the dark. Following fixation, 10 µL of PI was introduced. Finally, the stained cells were transferred to sterile vial and analyzed using a FACSCalibur flow cytometer (BD Biosciences, San Jose, CA, USA). Cell cycle distribution was assessed by the FlowJo software, which analyzed cells across the G1, S, and G2 phases. Data acquisition was conducted using three distinct sets of experiments.

### Detection of apoptosis by Annexin V FITC‑7AAD

To evaluate the percentage of cells undergoing apoptosis and necrosis triggerd by Chr and Qur, flow cytometry with 7-AAD and PE-annexin-V double staining was used. A549 cells (3 × 105 cells/well) were seeded in a 6-well plate and incubtated overnight before experiments. Cells were then exposed to IC₅₀ concentrations of free Chr, free Qur, Qur -Chr, Qur NPs, Chr NPs, and Chr- Qur @MSNs-Chi NPs for 72 h in a humidified incubator containing 5% CO_2_ at 37 °C. Prior to analysis, the cells were thoroughly rinsed with PBS, digested using trypsin, and then togethered by centrifugation. Afterward, the cells were rinsed twice with PBS, resuspended in 150 µL of annexin binding buffer, and stained with a combination of 10 µL of 7-AAD and PE-annexin-V solution. The stained cells were primarily incubated in the dark at at 25 °C for 15 min, followed by analysis using flow cytometry. Information was gathered from a gated population of 10,000 cells, and apoptotic and necrotic populations were conducted using Flowjo version 10.8.

###  In vitro scratch assay

The migration and proliferation abilities of A549 cells were assessed by an in vitro scratch wound healing experiment. A549 cells were grown in a 24-well plate at a density of 3 × 10^5^ cells/ml and incubated overnight in 10% FBS-RPMI-1640 medium at 37 °C and 5% CO_2_. A wound scratch line was created by employing a sterile 100 µL pipette tip when the cell confluency reached 90%, approximately. The scratched cell monolayer was exposed to IC_50_ concentrations of Free Chr, free Qur, Qur -Chr, Qur NPs, Chr NPs, and Chr- Qur @MSNs-Chi NPs, followed by a 24 h incubation. The progress of healing in the treated cells was detected using an inverted phase-contrast microscope (Leica, Germany) outfitted with a CCD camera, which recorded three representative images of the scratched area on each coverslip. This allowed for the assessment of cell migration under different conditions. Images of the wounds in the treated groups were captured at multiple time intervals, and the wound size was evaluated using ImageJ with a 20X objective. The initial results were documented in square pixels and then converted into the appropriate area units, allowing for the calculation of the wound closure percentage as outlined below:

$$\:\%\:of\:wound\:closure=\frac{(At\:=\:0h)\:-\:(At\:=\:24h)}{(At\:=\:0h)}\:\times\:100$$

At = 0 h: The measurement of the wound area was conducted right after the scratch was made.

At = 24 h: The measurement of the wound area was performed 24 h after the initial scratch.

### Real‑time PCR

To study the expression of apoptosis-related genes in A549 cancer cells treated with free and nanofurmoalte form of drugs, the culture medium was absolutely eliminated 24 h after incubating the cells with Free Chr, free Qur, Qur -Chr, Qur NPs, Chr NPs, and Chr- Qur @MSNs-Chi NPs. The cells were then rinsed with PBS and isolated separately. Following drug exposure, total RNA was extracted using the Trizol reagent, according to the instructions provided by the manufacturer. RNA quantity and quality were determined using a spectrophotometer (NanoDrop Technologies, USA) and 1.5% agarose gel electrophoresis, respectively. Total RNA was reverse transcribed using the cDNA synthesis kit (Yekta Tehiz Azma Company, Iran) according to the manufacturer’s instructions. After reverse transcription, the cDNA produced by SYBR Green PCR Master Mix (Ampliqon, Denmark) was used to analyze the expression of apoptosis- and cell cycle–related genes, including p53, Bax, Fas, Cyclin D1, pRB and Bcl2 by Real-time PCR. Relative quantification (ΔΔCt) values were then computed by standardizing the expression of the target gene against GAPDH levels. The findings were reported as fold change relative to the untreated control group.

### Statistical analysis

The outcomes are stated as the mean ± SD from at least 3 of three independent experiments. Statistical analyses were done using GraphPad Prism 6 software (GraphPad Software, Inc., La Jolla, CA). To evaluate significant differences among the study groups, one-way analysis of variance (ANOVA) was applied. Significance levels are indicated as *p < 0.05, **p < 0.01, ***p < 0.001, and ****p < 0.0001.

## Results

### Characterization of Dox-Chr-NPs

#### Measurements of particle size distribution and zeta potential

**Fig. 2:**
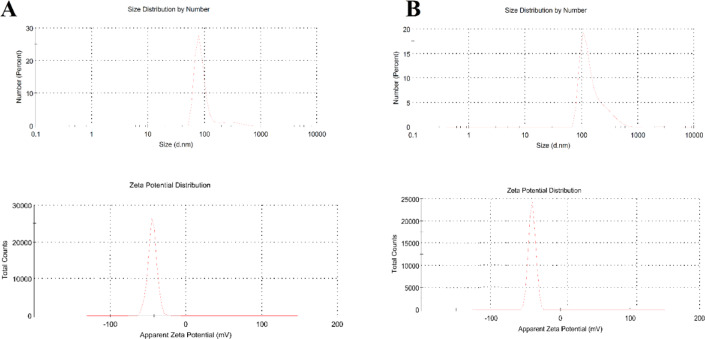
Dynamic light scattering (DLS) analysis of bare MSNs and Qur–Qur@MSNs–Chi nanoparticles shows their average hydrodynamic diameter, PDI, and ζ potential. The observed increase in particle size along with a shift to a positive ζ potential indicates that the chitosan coating was successfully applied, resulting in enhanced dispersion stability.

The characterization results of the MSNs-Chi NPs and Chr-Qur @MSNs-Chi NPs are summarized in Table 1. DLS analysis showed a uniform dispersion of MSNs-Chi NPs, with NPs an average size of 100.12 ± 5.5 nm, a PDI of 0.625 ± 0.199, and a ζ potential of − 24.3 mV for MSNs-Chi NPs. As shown in Fig. 2, the Chr-Qur @MSNs-Chi NPs revealed an average hydrodynamic diameter of 110.43 ± 2.3 nm, accompanied by a narrower PDI (0.295 ± 0.085)and a ζ potential of − 36.5mV, signifying enhanced colloidal stability following Chr and Qur encapsulation within the MSNs–Chi matrix.

**Table 1:** Particles’ sizes and zeta (ζ) potential of fabricated Chr-Qur @MSNs-Chi NPs results means ± SD (*n* = 3).

Nanomaterials	Mean diameter (nm)	Zeta potential (mV)	PDI
MSNs-Chi NPs	100.12 ± 5.5	− 24.3	0.625 ± 0.199
Qur MSNs-Chi NPs	108.17 ± 3.4	− 28.6	0.482 ± 0.113
Chr MSNs-Chi NPs	110.13 ± 6.3	− 28.5	0.225 ± 0.019
Chr- Qur @MSNs-Chi NPs	110.43 ± 2.3	− 36.5	0.295 ± 0.085

#### X-Ray diffraction (XRD) analysis

**Fig. 3:**
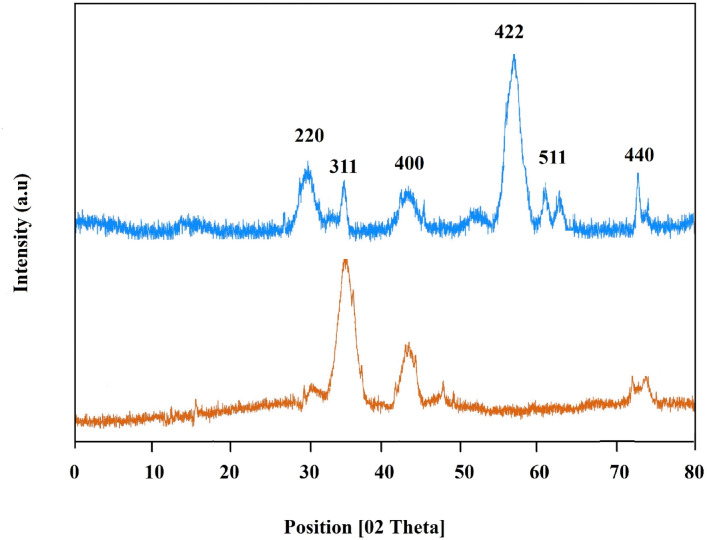
X-ray diffraction (XRD) patterns of bare MSNs–Chi nanoparticles and Chr–Qur@MSNs–Chi nanoparticles show the characteristic peaks of the mesoporous silica framework in both cases, indicating that the ordered structure of the silica is maintained even after drug loading and chitosan coating. Minor changes in peak intensity or broadening suggest the successful incorporation of the drug into the nanoparticles.

The XRD analysis verified the crystalline structure of the MSNs-Chi NPs. The XRD pattern of both MSNs-Chi NPs and Chr-Qur@MSNs-Chi NPs is presented in Fig. 2. The results revealed three Distinct sharp peaks within the 2θ range of 30–75°, corresponding to the (220), (311), and (400) diffraction lines. In the case of the Chr-Qur@MSNs-Chi NPs, sharp peaks were detected at 2θ values of. 35.1°, 55.5°, and 75°, which signify the characteristic reflections of the MSNs–Chi NPs framework, approving that drug encapsulation did not meaningfully change the crystalline nature of the NPs^20^ (Fig. 3).

#### BET surface area and pore structure analysis

**Fig. 4:**
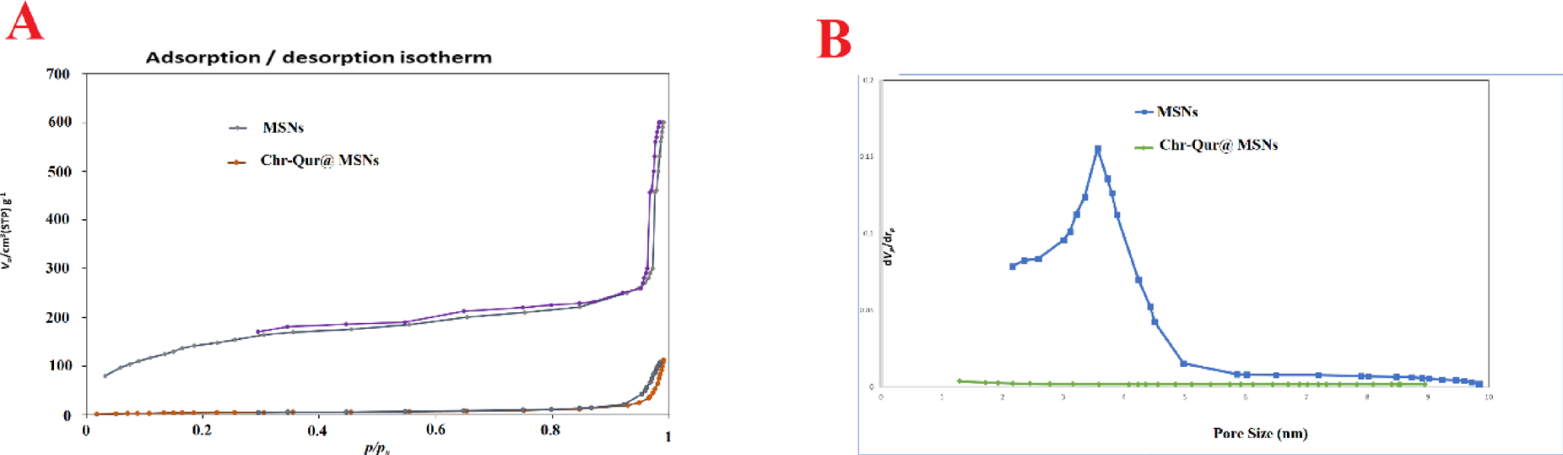
Mesoporous characteristics of MSNs. (**A**) Nitrogen adsorption–desorption isotherms of MSNs and Chr–Qur@MSNs–Chi NPs, illustrating the adsorption capacity and type of porosity of the materials. (**B**) Corresponding BJH pore size distribution plots, showing the pore size range, average pore diameter, and relative pore volume, highlighting differences between the bare and Chr–Qur@MSNs–Chi NPs.

BJH and BET analyses were done to assess the specific surface area and pore size distribution of the MSNs (Fig. 4). Consistent with the IUPAC classification, the MSNs showed a typical permanent type IV isotherm, which is specific of mesoporous materials. The unloaded MSNs had a BET surface area of 610 ± 10.4 m²/g, a pore volume of 0.62 ± 0.12 cm³/g, and an average pore diameter of 4.42 ± 0.5 nm. Following Chr and Qur loading, these parameters decreased, with Chr-Qur@MSNs-Chi NPs showing a surface area of 97.1 ± 3.4 m²/g, a pore volume of 0.42 ± 0.13 cm³/g, and a pore diameter of 1.85 ± 0.3 nm, indicating effective drug encapsulation within the mesoporous framework.

#### Morphological analysis of MSNs–Chi NPs

**Fig. 5:**
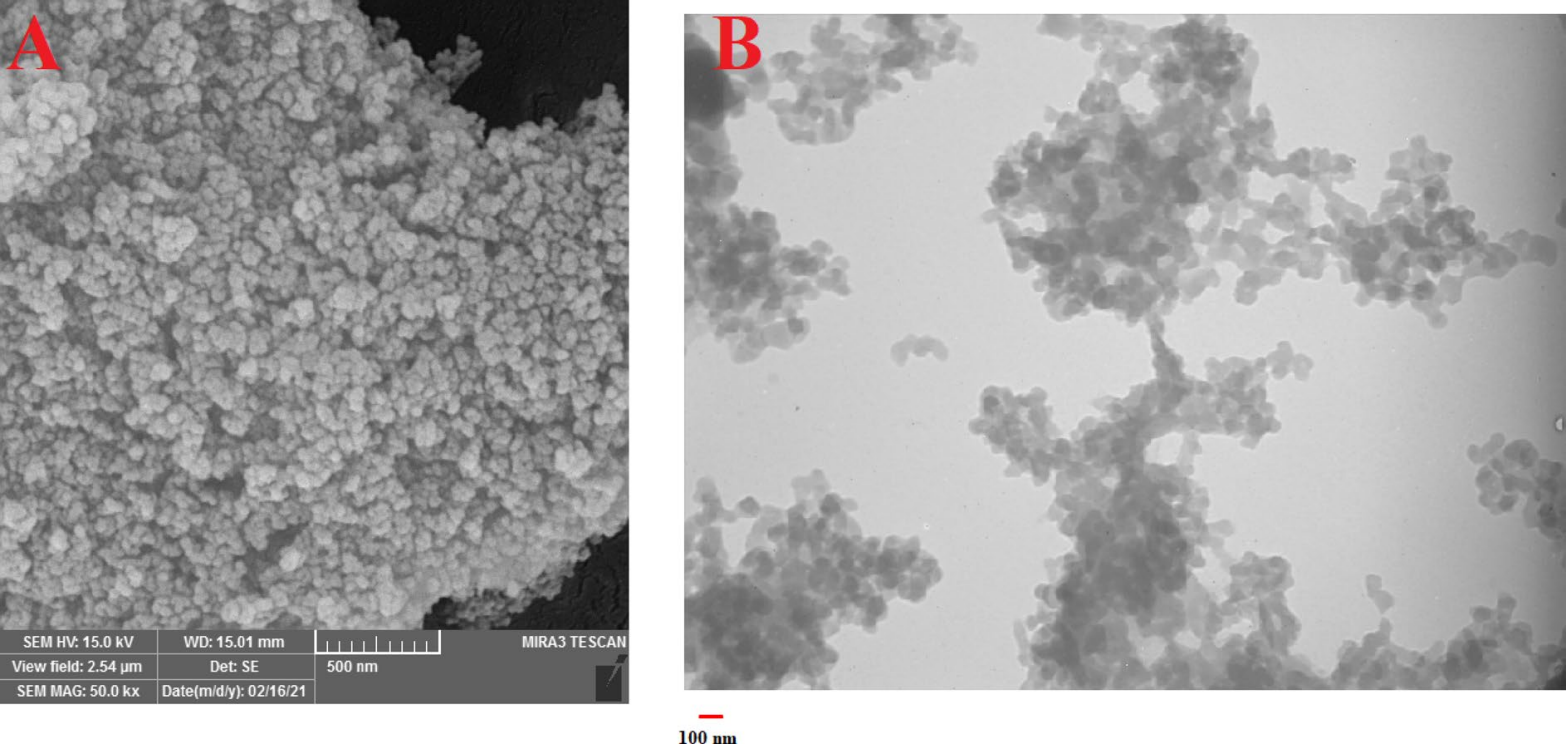
Morphological characterization of Chr–Qur@MSNs–Chi nanoparticles. (**A**) Scanning electron microscopy (SEM) images showing the surface morphology and uniform spherical shape of the nanoparticles. (**B**) Transmission electron microscopy (TEM) images revealing the internal mesoporous structure and successful incorporation of Chr–Qur within the silica framework. Both SEM and TEM images confirm the preservation of the mesoporous structure after chitosan coating and drug loading.

The characterization of MSNs-Chi NPs surface appearance, size, and structural characteristics was further studied using FE-SEM and TEM. As observed in the FE-SEM image, Chr-Qur @MSNs-Chi NPs exhibited even shape and spherical nature with a size of 100 nm (Fig. 5A). The TEM micrographs specify that the particle sizes were around 110 nm, presenting a spherical morphology. The particle size measured by TEM closely corresponded with the hydrodynamic diameter obtained from DLS analysis, validating the consistency of the characterization results (Fig. 5B).

#### FTIR analysis

**Fig. 6:**
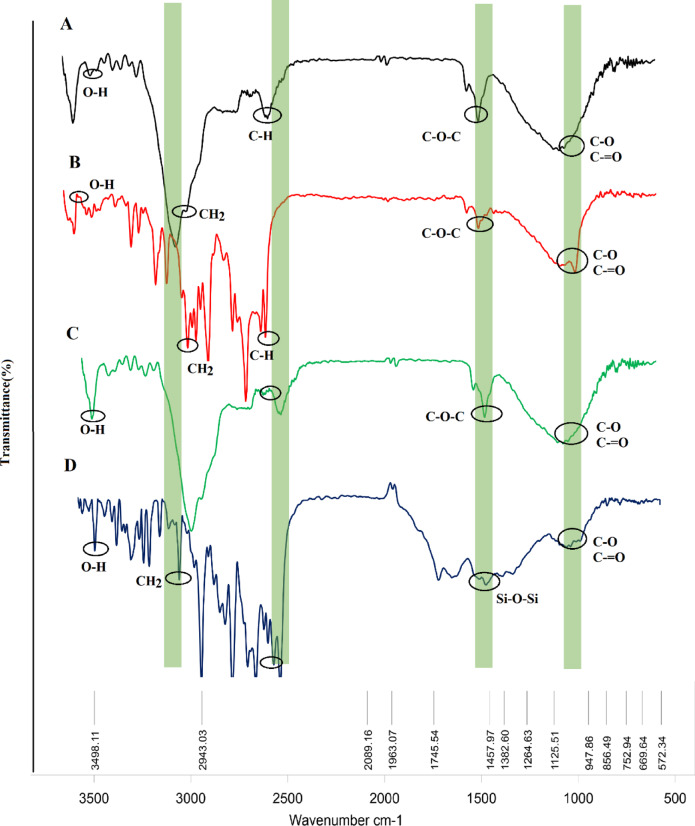
Fourier-transform infrared (FTIR) spectra of the materials are shown for (**A**) Chr, (**B**) Qur, (**C**) a physical mixture of Chr–Qur, and (**D**) Chr–Qur@MSNs–Chi nanoparticles. The spectra display the characteristic functional group vibrations of Chr and Qur, while the shifts and appearance of new peaks in the Chr–Qur@MSNs–Chi sample confirm successful drug loading and interactions with the chitosan-coated mesoporous silica framework.

The FTIR analysis gives specific visions into the compositions, structural elements, and interactions of the studied compounds. FT-IR analysis was performed on Qur, Chr, MSNs-Chi NPs, and Chr-Qur @MSNs-Chi NPs, demonstrating the incorporation of Qur and Chr within the MSNs-Chi NPs (Fig. 6). The spectrum of unbound Qur exhibits broad peaks at 3200 cm^− 1^, attributed to the O–H bonds present in the drug. A prominent peak observed around 1435 cm^− 1^ signifies the presence of the O–C = O bond within the NPs^20,21^. For Chr-Qur @MSNs-Chi NPs, the presence of all distinctive peaks associated with both MSNs and Chr- indicates the successful integration and loading of Chr and Qur into the nanoparticles. This variance confirms that the loading of Chr and Qur in n MSNs-Chi NPs has been executed accurately.

### Assessment of DL and EE

The evaluations of EE and DL confirmed the effective incorporation of both Qur and Chr into the MSNs-Chi NPs. The investigation unveiled that the integration of Qur and Chr into the NPs yielded encapsulation efficiencies of 90.38 ± 0.1% with coreesponding drug loading capacities of 13.2 ± 4.3%, demonstrating the strong affinity of the drug molecules for the MSNs–Chi matrix and the efficiency of the encapsulation procedure.

### In vitro drug release profiles

**Fig. 7:**
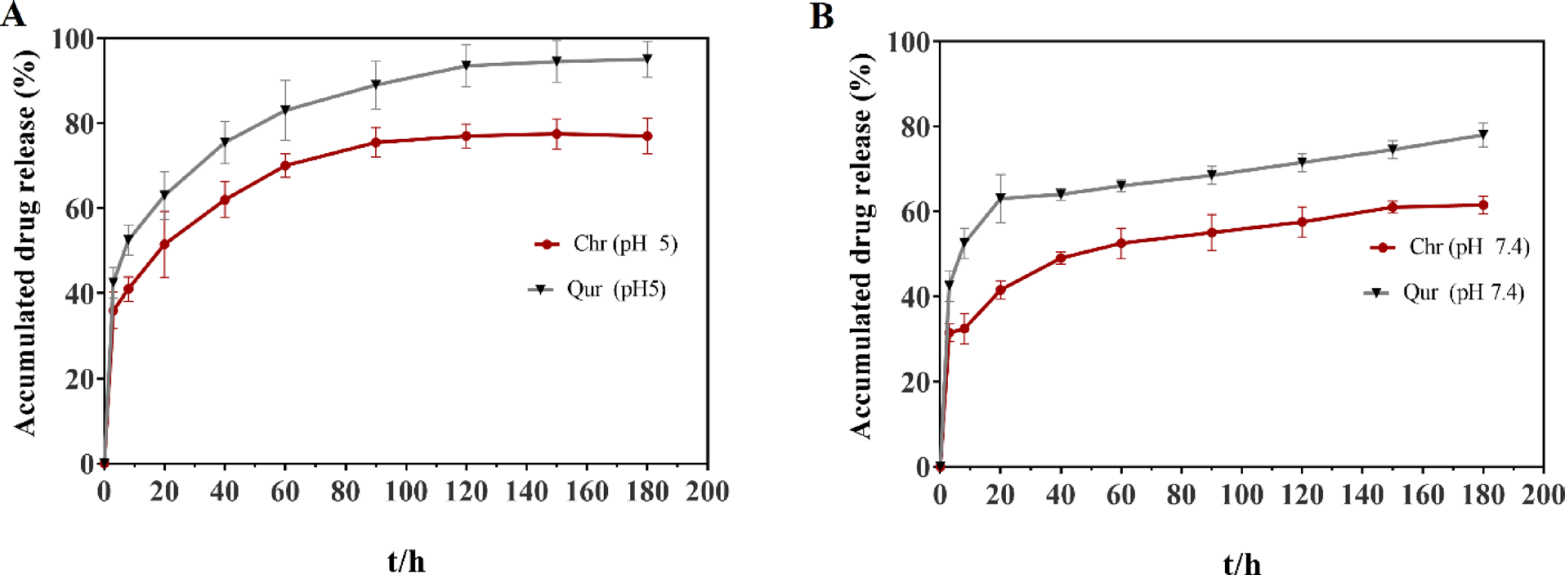
In vitro release profiles of Qur and Chr from Chr–Qur@MSNs–Chi nanoparticles were evaluated at pH 5.0 (**A**) and pH 7.4 (**B**). The cumulative release over time is presented as mean ± standard deviation (*n* = 3). The results show pH-dependent behavior, with a faster release under acidic conditions, suggesting the nanoparticles could provide targeted drug delivery in the acidic tumor microenvironment.

The dialysis membrane technique was used at pH 7.4 and 5.0 to study the discharge profile of Qur and Chr from the MSNs-Chi NPs, to mimic physiological and tumor microenvironments, respectively. The Fig. 7 shows the in vitro drug release of Chr-Qur @MSNs-Chi NPs. The NPs exhibit a sustained release pattern, with a notably faster drug release rate observed at pH 5.0 ( tumor microenvironment) compared to pH 7.4 (rphysiological condition). This accelerated release under acidic conditions can be attributed to feeble electrostatic interactions between the drug molecules and the NPs matrix, as well as the faster degradation of the chitosan coating at low pH.

At pH 5, the release rate was faster, with 95% and 75% of Que and Chr being released over 200 h. Both drugs exhibited similar biphasic discharge behavior, characterized by an initial burst phase followed by a prolonged sustained release period. The amount of Chr released within the initial 48 and 72 h closely resembled that of Qur, with a gradual decline observed after 72 h, eventually reaching lower concentrations. Furthermore, it was revealed that both Qur and Chr could be released simultaneously, marked by an initial phase of rapid release, succeeded by a slower release of the drugs. The notable distinction in the drug release pattern among Chr-Qur @MSNs-Chi NPs is due to variances in their hydrophobicity, physicochemical characteristics, and drug distribution within the NPs. Additionally, the drug release studies reveal the strong stability of drug molecules bound by electrostatic interactions at physiological pH, while release is prompted in acidic conditions. To further elucidate the release mechanism, the experimental data were fitted to various kinetic models (Table 2). The correlation coefficient (R²) value were used to determine the most appropriate model for each formulation. The formulation with the highest R² value was picked as the best model for the release mechanism. The Higuchi model had the strongest correlation (R² = 0.986), suggesting that the drug release is primarily controlled by diffusion through the polymer matrix. Additionally, the release exponent (*n* = 0.46) obtained from the Korsmeyer–Peppas model supports that the release follows a Fickian diffusion mechanism.

**Table 2:** The mathematical models correlation coefficients and release exponents of Chr and Qur from Chr- Qur @MSNs-Chi NPs.

		Zero order	First order	Higuchi model
Kinetic models		Ct = C0 + K0t	LogC = LogC0 + Kt/2.303	Q = KH √t
		R^2^	R^2^	R^2^
Qur @MSNs-Chi NPs	pH = 5	0.819	0.98	0.958
Chr @MSNs-Chi NPs	pH = 5	0.7635	0.7932	0.7635
Chr- Qur @MSNs-Chi NPs	pH = 5	0.4563	0.4963	06324
Qur @MSNs-Chi NPs	pH = 7.4	0.835	0.8352	0.8793
Chr @MSNs-Chi NPs	pH = 7.4	0.7163	0.9536	0.7393
Chr- Qur @MSNs-Chi NPs	pH = 7.4	0.6182	0.6162	0.6793

### Cytotoxicity over erythrocytes

**Fig. 8:**
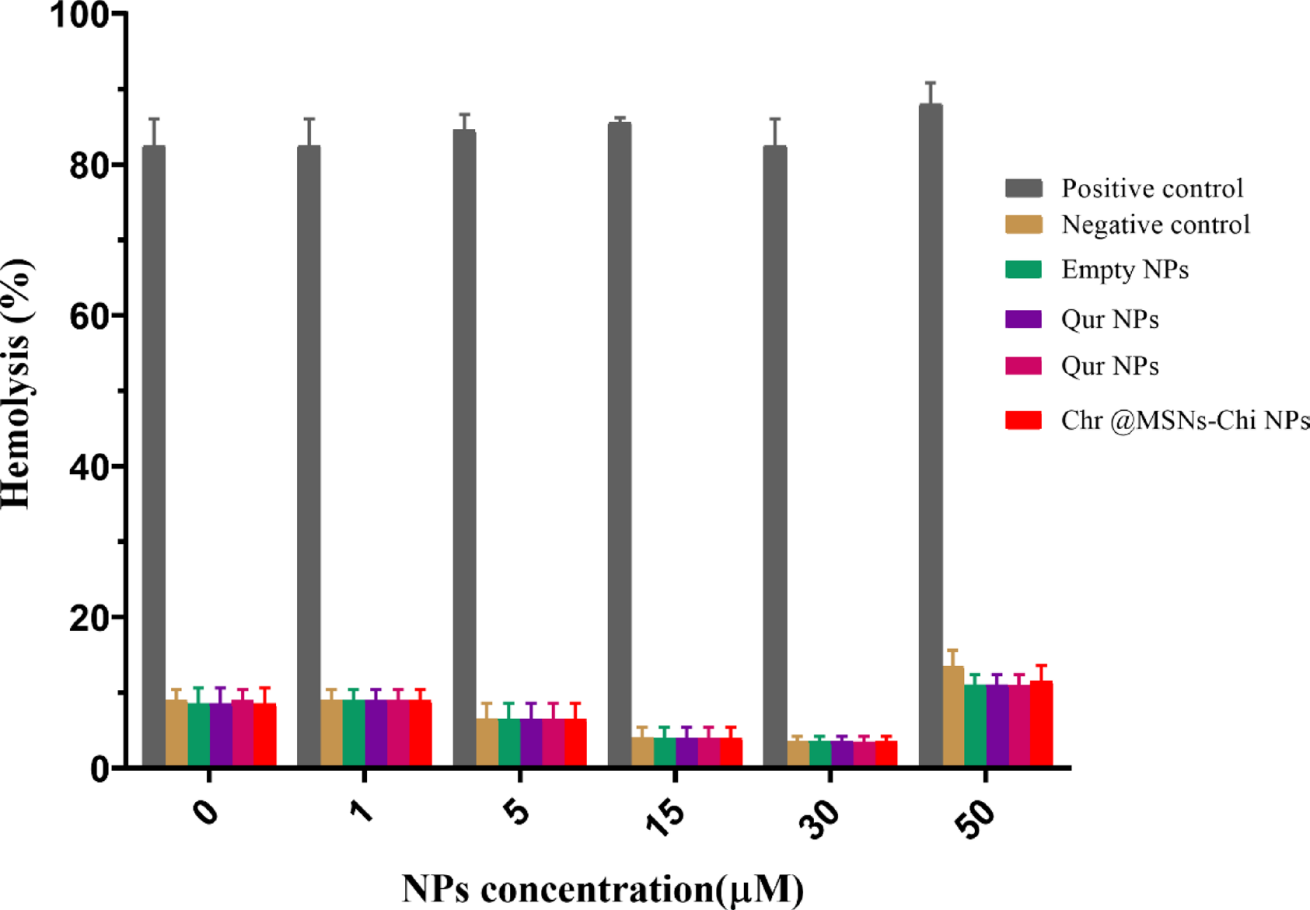
Percentage hemolysis caused by the positive control (Triton X-100), negative control (PBS), free Chr, free Qur, the Chr–Qur mixture, individual nanoparticles (Qur NPs, Chr NPs), and Chr–Qur@MSNs–Chi nanoparticles. Data are shown as mean ± SD (*n* = 3).

The release of hemoglobin from erythrocytes was utilized to evaluate the potential of NPs to induce hemolytic activity. Both MSNs-Chi NPs and Chr-Qur@MSNs-Chi NPs confirmed good biocompatibility with RBC, displaying no significant hemolytic effects. As shown in Fig. 8. MSNs-Chi NPs dispaled 7% at the highest tested concentrations. Likewise, Chr-Qur@MSNs-Chi NPs exhibited good compatibility at concentrations up to 50 µM, with hemolysis rates of around 6%.

### Cell cytotoxicity

**Fig. 9:**
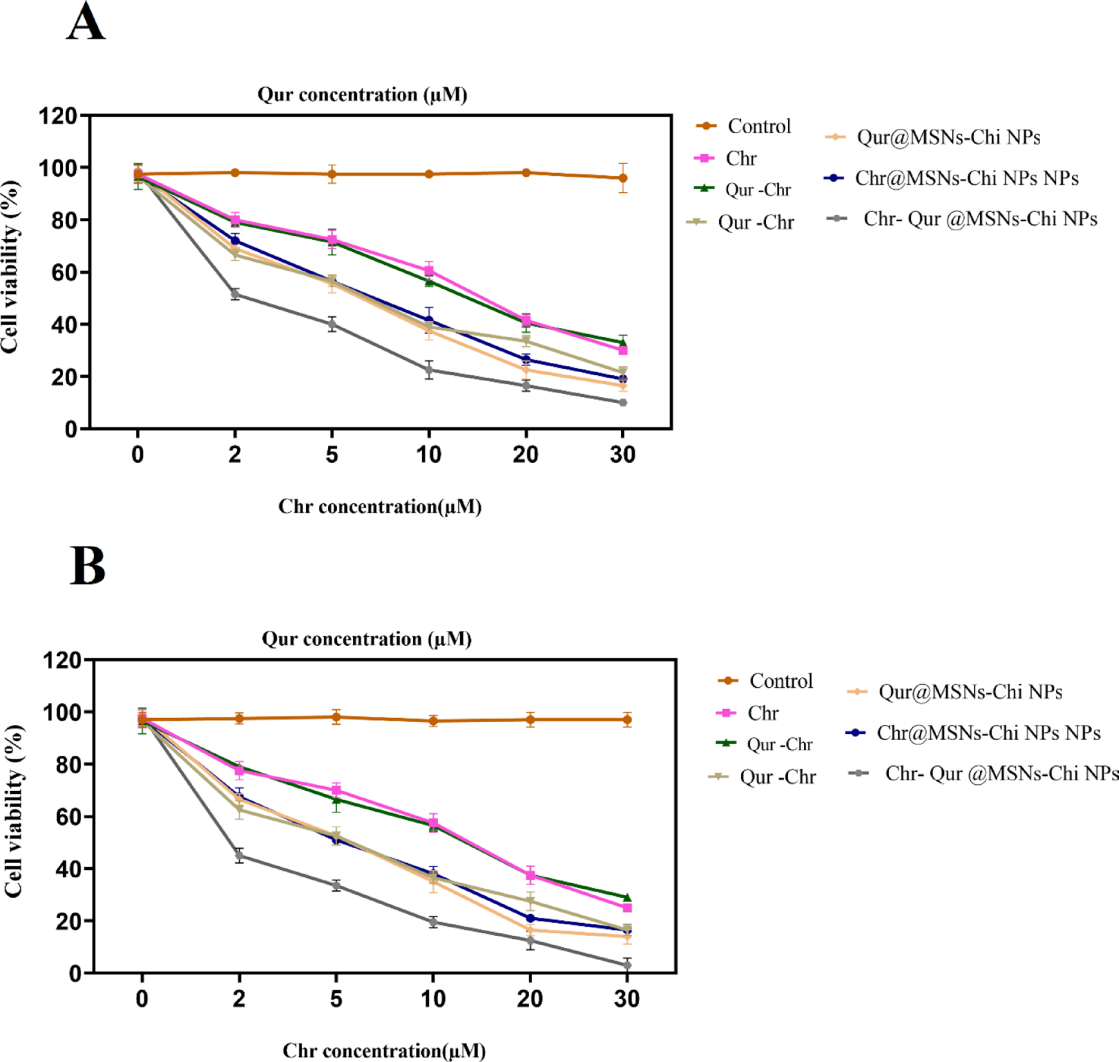
Percentage cell viability of A549 human lung cancer cells following treatment with freeChr, free Qur, their physical combination (Qur–Chr), Qur NPs, Chr NPs and Chr–Qur@MSNs–Chi NPs for (**A**) 24 h, (**B**) 48 h. Cell viability was assessed using the MTT assay. Results are expressed as mean ± standard deviation (SD) from three independent experiments (*n* = 3).

The cytotoxic effects of Qur NPs, Chr NPs, and Chr- Qur @MSNs-Chi NPs on A549 cancer cells were assessed using the MTT analysis. As illustrated in Fig. 9, exposure to different drug concentrations for 24 and 48 h resulted in a concentration-dependent inhibition of cell growth in both the free and nano-encapsulated versions of Qur and Chr. The IC_50_ values were determined from the MTT assay data using GraphPad Prism software (version 6.7). Therefore, simultaneous loading of Qur and Chr into MSNs-Chi NPs demonstrated enhanced cytotoxic effects compared to the free single drug form .Moreover, the cytotoxic induced by entrapped Qur and Chr was significantly greater than that of the corresonding free drugs (Table 3). Following 48 h of treatment, the IC_50_ values for Free Chr, free Qur, Qur -Chr, Qur NPs, Chr NPs, and Chr- Qur @MSNs-Chi NPs were 12, 11, 7, 7, 5, 1 µM, respectively. The enhanced growth inhibition effects, observed for the nano-encapsulated drugs is likely due to the regulated discharge of drugs from the NPs and the improved cellular uptake of the encapsulated compounds.

**Table 3:** Comparative IC50 values for A549 cell line treated with pure and nanocapsulation of each drug within 24 h and 48 h.

Anticancer agent	IC_50_ (µM)	
	24 h	48 h
Free Chr	15 ± 0.2 µM	12 ± 0.1 µM
Free Qur	12 ± 0.3 µM	11 ± 0.3 µM
Qur -Chr	8 ± 0.6 µM	7 ± 0.1 µM
Qur @MSNs-Chi NPs	8 ± 0.5 µM	7 ± 0.2 µM
Chr @MSNs-Chi NPs	7 ± 0.2 µM	5 ± 0.3 µM
Chr- Qur @MSNs-Chi NPs	2 ± 0.1 µM	1 ± 0.2 µM

*Dissimilar superscript symbols (within row) and letters (within column) indicate significance at *p* ≤ 0.05.

### Nuclear changes in A549 cells

**Fig. 10:**
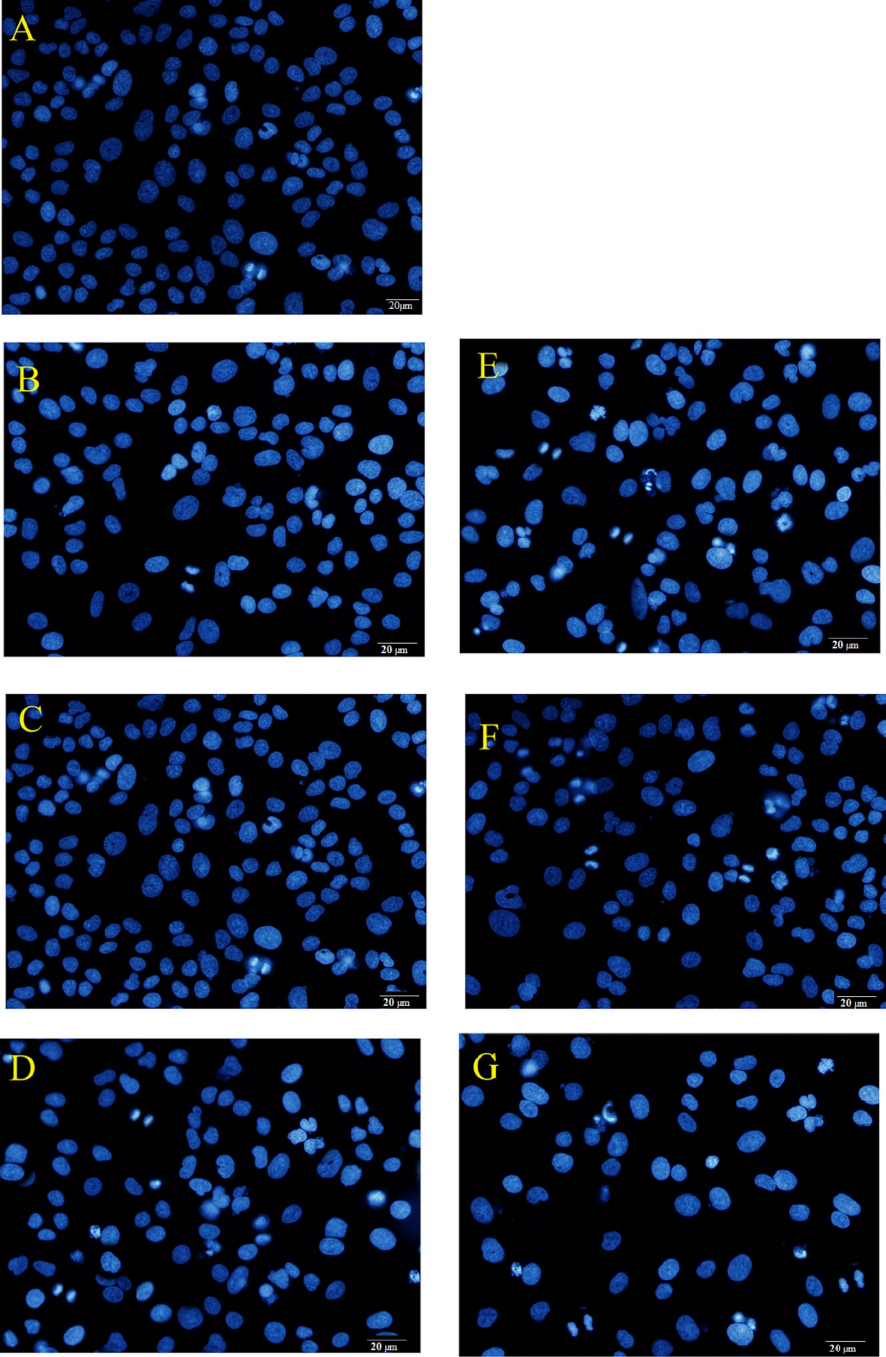
Fluorescence microscopy images showing nuclear morphology in A549 cells stained with DAPI. DAPI binds precisely to DNA, allowing clear visualization of nuclei and evaluation of nuclear integrity. Cells were treated under the following conditions: control (**A**), free Chr (**B**), free Qur (**C**), Chr–NPs (**D**), Qur–NPs (**E**), Chr–Qur (**F**), and Chr–Qur@MSNs-Chi NPs (**G**). Images were acquired using a 20× objective on a fluorescence microscope. Compared to the control group, treated cells exhibit noticeable changes in nuclear shape and organization, indicating the effects of the compounds and their nanoformulations on apoptosis and nuclear structure. Scale bars = 20 μm.

DAPI staining was done to determine whether the inhibition of cell proliferation in A549 cells treated with Chr-Qur @MSNs-Chi NPs resulted in nuclear fragmentation. The extent of DNA condensation in A549 lung cancer cells treated with Chr, Qur, Qur-Chr, Qur NPs, Chr NPs, and Chr- Qur @MSNsChi NPs was evaluated using DAPI nuclear staining, as illustrated in Fig. 10A-G. The findings revealed a marked increase in chromatin condensation and nuclear fragmentation in the cancer cells exposed to drog loaded MSNs-Chi NPs compared to those treated with free drugs and the untreated control group. In addition, the density of A549 cancer cells decreased more substantially in the group treated with Chr-Qur@MSNs-Chi NPs than in the group treated with single drug-loaded NPs. These finding supports the notion that Chr-Qur@MSNs-Chi NPs trigger apoptosis in A549 cells more efficiently than free drug formulations.

### Analysis of cell cycle arrest

**Fig. 11:**
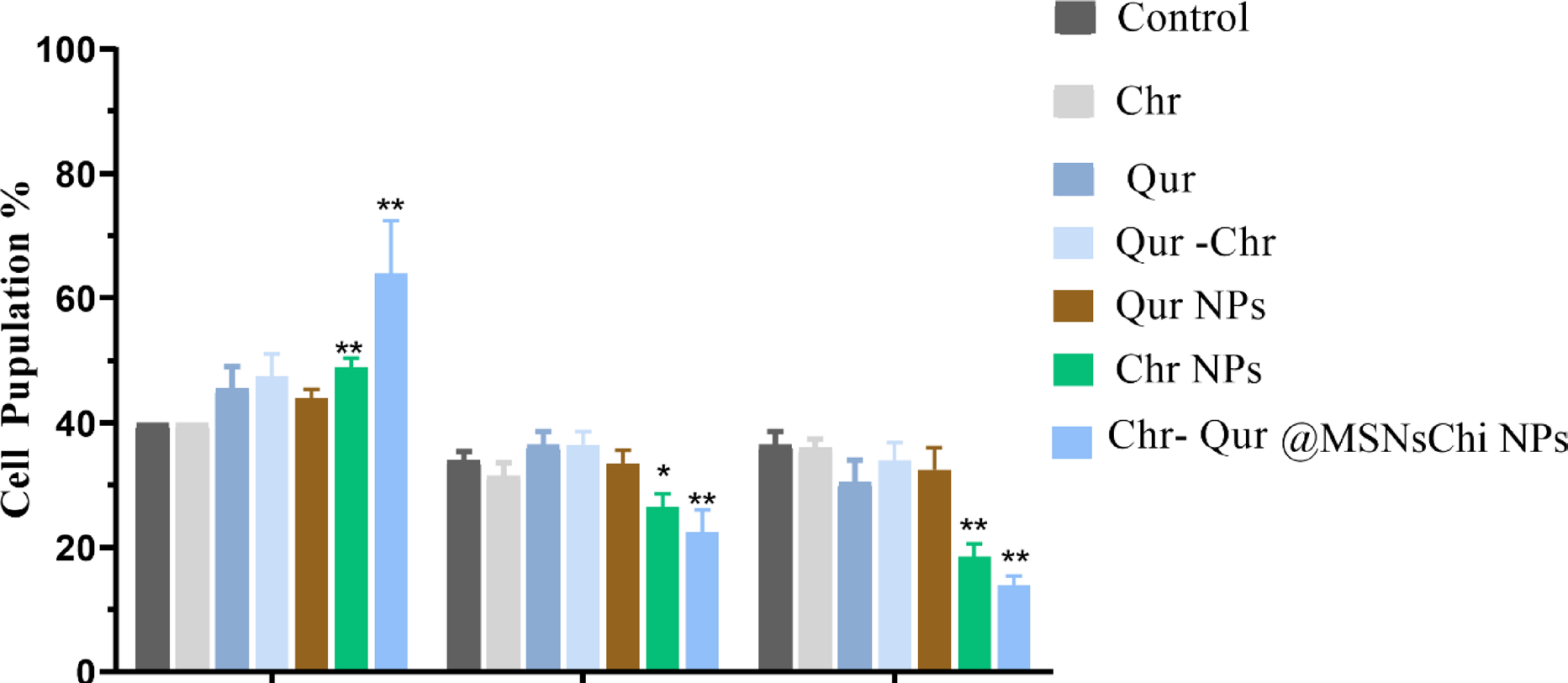
Cell cycle distribution was analyzed in A549 cells following 48-h exposure to the IC_50_ concentrations of free Chr, free Qur, Chr–NPs, Qur–NPs, Chr–Qur, and Chr–Qur@MSNs-Chi NPs, with untreated cells serving as controls. Data are presented as mean ± SEM (*n* = 3), with statistical significance indicated as **p < 0.01 and ***p < 0.005.

According to the cell cycle analysis, noticeable differences were observed among the treated groups. In the control group, A549 cells exhibited a typical distribution pattern, with 40% of cells in the G0/G1 phase, 35% in the S phase, and 35% in the G2/M phase, indicating normal proliferation (Fig. 11). Treatment with free Chr produced only minor changes, maintaining 40% of cells in G0/G1, 30% in S, and 35% in G2/M. Exposure to free Qur slightly increased the G0/G1 population to 48%, with 35% and 33% of cells in the S and G2/M phases, respectively. The combined free Qur–Chr treatment resulted in 45% of cells in G0/G1, 38% in S, and 36% in G2/M, suggesting a mild synergistic interaction. Similarly, Qur NPs caused minor changes, with 45% of cells in G0/G1, 35% in S, and 35% in G2/M. In contrast, Chr NPs led to a marked reduction in the S (25%) and G2/M (20%) phases and an increase in the G0/G1 phase to 48%. The most substantial effect was observed with Chr-Qur@MSNs-Chi NPs, where the G0/G1 population rose sharply to 70%, while the S and G2/M phases decreased to 20% and 15%, respectively (Fig. 11). This pronounced shift indicates a strong accumulation of cells in the sub-G1 phase, reflecting significant apoptotic activity induced by the nanoparticle formulation.

### Annexin V FITC/7AAD staining

**Fig. 12:**
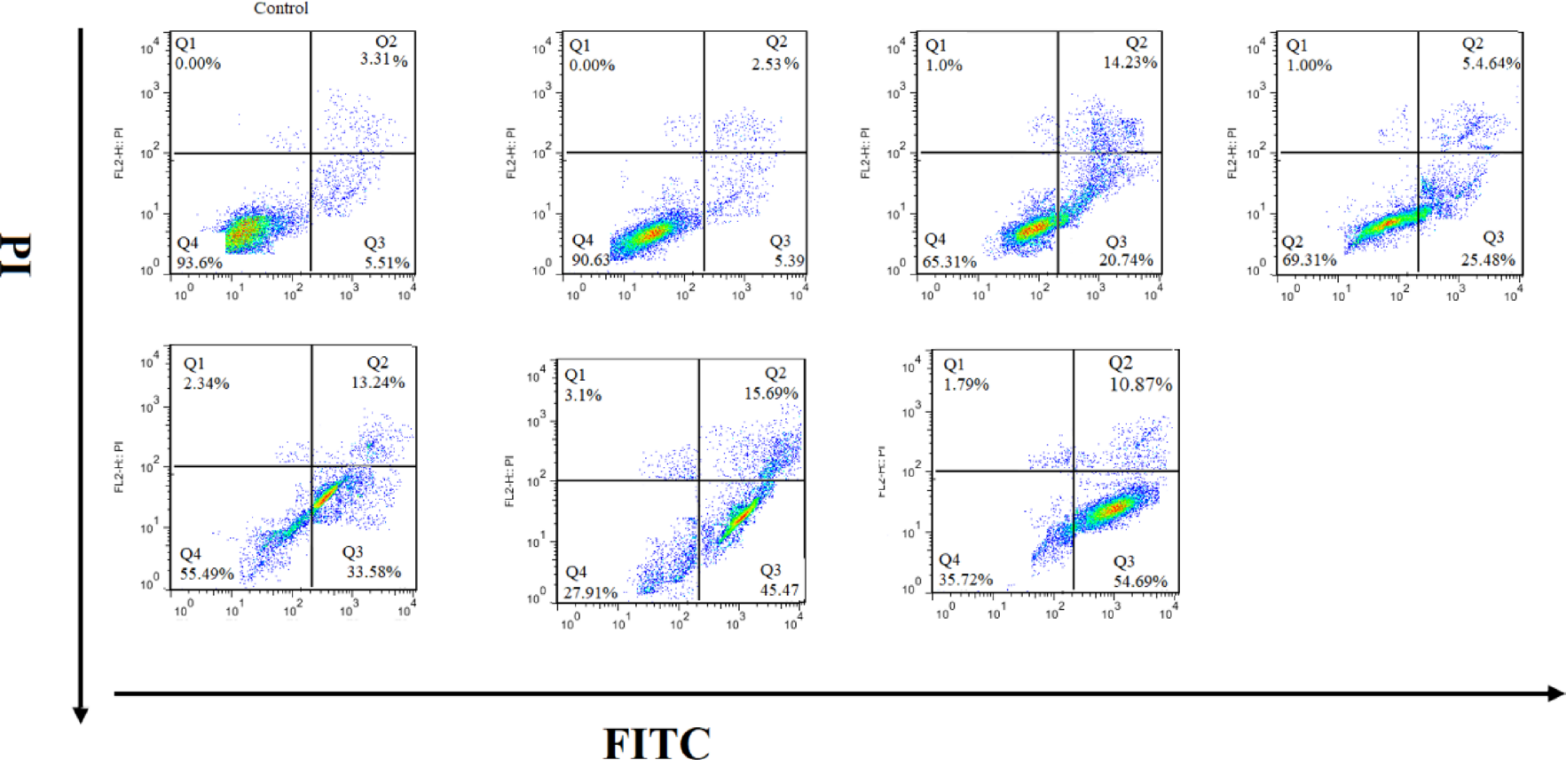
Flow cytometry was performed on A549 cells treated with control, free Chr, free Qur, Chr–NPs, Qur –NPs, Chr–Qur, and Chr- Qur@MSNsChi NPs for 48 h, followed by Annexin V FITC and 7AAD staining. The plot is divided into four quadrants representing different cell types: necrotic (Q1), late apoptotic (Q2), viable (Q3), and early apoptotic (Q4) cells. The percentage of cells in each category is shown within its respective quadrant.The results, derived from three independent experiments, are presented as mean ± SD (*n* = 3). Statistical significance was set at *p* < 0.05 versus the control, with **p ≤ 0.01 and ***p ≤ 0.001.

To better understand the molecular basis behind the growth-inhibitory effects of Chr, Qur, and Chr-Qur@MSNsChi NPs, we assessed the percentage of apoptotic and necrotic cells A549 cells through FITC-conjugated annexin V and PI staining. The representative dot plots clearly display the percentages of Q1 (necrotic), Q2 (late apoptotic), Q3 (viable), and Q4 (early apoptotic) cell populations in each quadrant. A clear difference in both early and late apoptosis rates was observed between the cells treated with free drugs and those treated with Chr-Qur@MSNsChi NPs. As shown in Fig. 12, treatment with free Chr, Qur, Qur -Chr, Qur NPs, Chr NPs, and Chr- Qur@MSNsChi NPs induced early apoptosis rates of 5.51%, 5.39%, 20.74%, 25.48%, 33.58, 45.37%, and 54.69%, respectively. These findings demonstrate that co-encapsulation of Qur and Chr within MSNs-Chi NPs noticeably enhances apoptosis in A549 cells, with a maximal apoptotic rate of 54.69% observed in cells treated with Chr–Qur@MSNs-Chi NPs.

### Cell migration assay

**Fig. 13:**
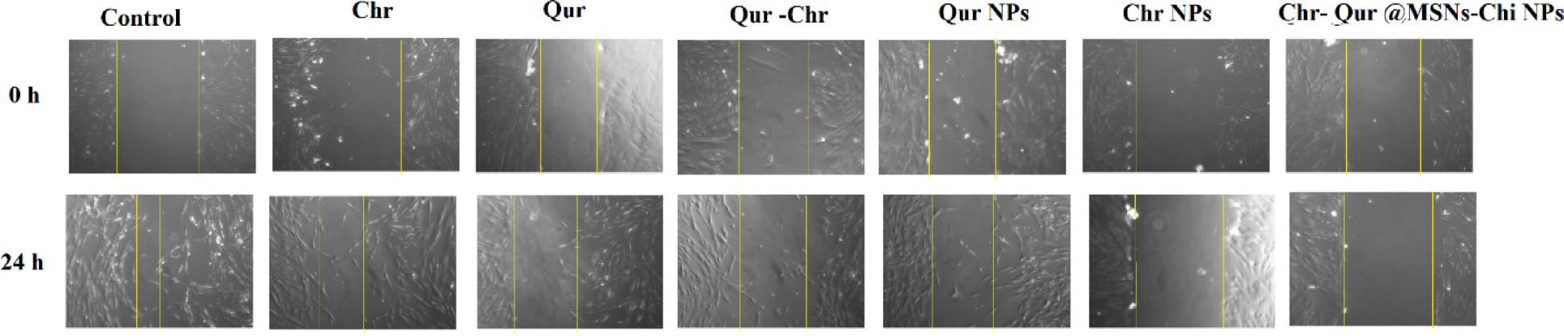
The impact of Chr and Qur on the migration ability of lung cancer cells was assessed using a wound healing assay. The results demonstrated a significant reduction in the migration of Chr- Qur@MSNsChi NPs treated A549 lung cancer cells compared to controls. Representative images and quantitative analysis revealed a lower number of migrated cells per field, highlighting the diminished migratory and invasive capabilities following treatment with Qur@MSNsChi NPs. Statistical analysis, conducted using the Student’s t-test, confirmed the significance of these findings (*P < 0.05, ***P < 0.001, ****P < 0.0001). Images were taken with a 20 ×lens.

A scratch wound healing assay was performed to assess the effects of Qur, Chr, and Qur -Chr in both their free and nano-encapsulated forms on the migration of A549 cells. The wound width and closure distance were measured using image analysis software over a 24 h period, with comparisons made between images taken at 0, and 24 h. In the control group, A549 cells migrated to cover approximately 50% to 60% of the initial wound area within 24 h. In contrast, treatment with Chr NPs, Qur NPs, and Chr-Qur@MSNsChi NPs significantly reduced cell migration compared to the untreated group. The wound edges were marked to track the progression of closure throughout the experiment. The experiments were performed in triplicate, and the results are presented as mean ± standard error. After 24 h, both Qur NPs and Chr-NPs, as well as Chr-Qur@MSNsChi-NPs, significantly inhibited A549 cells migration relative to the control group. Notably, Chr-Qur@MSNsChi NPs showed a particularly strong reduction in A549 cell migration. These results suggest that encapsulation Chr-Qur in MSNsChi NPs enhances their anti-migratory potential, as illustrated in Fig. 13.

### Examining gene expression

**Fig. 14:**
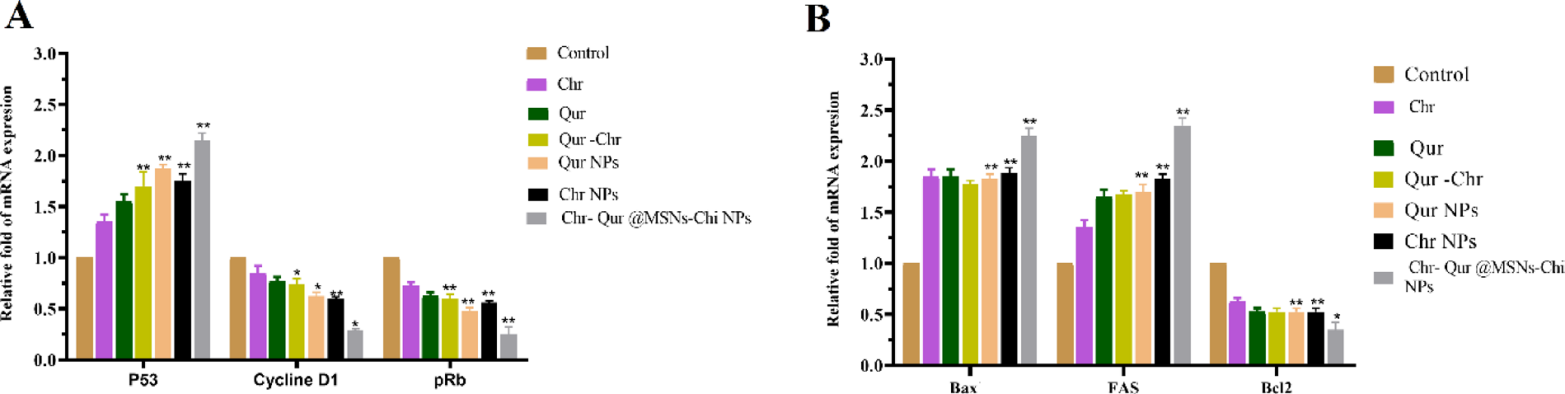
Analysis of apoptosis-related gene expression in A549 cells following treatment with Qur and Chr, delivered either in free form or as nanoformulations. (A) The mRNA expression of anti-apoptotic genes (Rbp, Cyclin D1, and Bcl2) was markedly decreased in the treated groups compared with the control. (B) Conversely, the expression of pro-apoptotic genes (Bax, Fas, and P53) was significantly increased after treatment with control, free Chr, free Qur, Chr–NPs, Qur –NPs, Chr–Qur, and Chr-Qur@MSNsChi NPs for 48 h. Error bars represent standard deviations. (*P value < 0.05, **P value < 0.001).

In the final stage, the in vitro evaluation focused on assessing the expression of a small subset of genes associated with apoptosis. In contrast to the control group, both the treatment groups in free or nanoformulated forms, notably suppressed the expression of Rbp, Cyclin D1, and Bcl2, which are key regulators of cell survival and cell cycle progression. (Figure 14A and B). Additionally, the treatment group exhibited a significant upregulation in the expression levels of pro-apoptotic genes, including Bax, Fas, and P53, as illustrated in Fig. 14B. These findings suggest that Qur and Chr hinder the growth of A549 cells by regulating the expression levels of both pro-apoptotic and anti-apoptotic genes.

## Discussion

Although conventional chemotherapy remains a cornerstone of cancer management, it faces major challenges such as severe systemic toxicity and poor drug bioavailability, largely resulting from its high cytotoxicity^22,23^. These limitations have driven the search for alternative therapeutic strategies. Recent research indicates that the combination of two or more therapeutic compounds can produce synergistica effects in cancer treatment. This strategy not only helps mitigate side effects but also enhances patients’ quality of life. By simultaneously targeting multiple pathways involved in cancer progression, these approaches have demonstrated effectiveness in overcoming drug resistance, inhibiting cancer cell growth, and reducing the risk of metastasis^24,25^.

Consistent with previous reports, both Chr and Qur showed substantial anti-proliferative and pro-apoptotic effects in A549 cells^10^. Moreover, Chr and Qur have been shown to increase the sensitivity of lung cancer cells to conventional anticancer medications, indicating their potential use in combination therapy to enhance treatment outcomes for lung cancer patients^26,27^. Notably, the combination of these compounds demonstrated synergistic effects, leading to enhanced inhibition of cell growth and higher apoptotic induction compared to single-agent treatments^28^. Despite their promising anticancer effects, the clinical translation of Chr and Qur remains limited due to their poor water solubility and rapid metabolism, which hinder effective drug delivery and therapeutic efficacy^29^. Thus, research focusing on improving their solubility, stability, and targeted delivery is crucial to fully realize their therapeutic potential^30^. To address the challenges outlined above, particularly NPs-based delivery systems MSNs, have emergedas as a promising solution for enhancing drug delivery systems. MSNs represent a versatile drug delivery platform, characterized by their biodegradability, biocompatibility, non-immunogenicity, and favorable safety profile^18^. Abouaitah et al. discovered folic acid-conjugated MSNs as carriers for anticancer prodrugs, including Qur, aiming for targeted delivery to cancer cells. Their results confirmed better cellular uptake and continued drug release, highlighting the potential of functionalized MSNs in improving the therapeutic effectiveness of natural compounds^31^.

In the present study, a high drug entrapment efficiency of approximately 90.38% was achieved, indicating successful encapsulation of both Qur and Chr. These findings are align with previous reports on MSNs prepared using similar techniques, demonstrating consistency across various types of MSNs^32^. The physicochemical properties of Chr-Qur@MSNsChi NPs suggest that this system serves as cost-effective, robust, and highly penetrative vehicle for transporting Qur and Chr to cancer cells. Dhingra et al. highlighted the potential of MSNs as efficient drug delivery vehicles in tumor treatment. Their biodegradability, promising biodistribution, and acquiescence to surface modifications make MSNs mainly well-suited for targeted approaches, locating them as a hopeful platform for lung cancer treatment^33^.

In comparison to the free administration of Qur and Chr, the Chr-Qur -NPs exhibited heightened cytotoxicity against A549 cells. Consistent with earlier studies, the synergistic combination of Qur and Chr within the nanoparticle matrix significantly enhanced anti-tumor efficacy compared to their individual or free forms^34^.

An analysis of particle sizes revealed that the incorporation of Qur and Chr within the MSNsChi NPs led to an increase in the volume of the NPs, resulting in larger dimensions compared to empty MSNsChi NPs. Specifically, the Qur-Chr-loaded MSNsChi NPs exhibited the largest mean diameter and the lowest PDI, suggesting that the nano-encapsulation of the drugs contributed to improved uniformity and stability of the formulation.

The surface charge of NP emulsions significantly affects both their short- and long-term stability, as well as their interactions with cell membranes^35^. It has been observed that NPs with smaller average sizes tend to exhibit higher zeta potential values values than larger ones. This phenomenon can be attributed to the faster electrophoretic mobility of smaller NPs in an electric field, resulting in enhanced zeta potential values and, consequently, enhanced the stability of NPs in colloidal dispersions. Previous studies have reported that most cancer cells preferentially internalize NPs sized below 400 nm^36^. Moreover, for successful endocytosis, NPs need to be sufficiently large to prevent quick leakage into capillaries and to evade clearance by macrophages^37^. Considering this physicochemical attributes, the Qur-Chr-loaded MSNsChi NPs appear to be well-suited for efficient cellular uptake.

These NPs exhibit strong potential for the concurrent transport of hydrophobic phytochemicals. Particularly, their pH-sensitive release manners permits them to specially release Qur and Chr in acidic environments, such as the tumor microenvironment or intracellular compartments like endosomes and lysosomes. This targeted discharge can rise the drugs’ accessibility inside cancer cells while possibly decreasing off-target effects, so increasing therapeutic precision^38^. Drug release was faster at acidic pH (5) than at physiological pH (7.4), consistent with the intended pH-responsive design. Some kinetic models showed low R² values, indicating that release is governed by multiple processes. Overall, the release mechanism likely involves both diffusion and chitosan^39,40^.

In the MTT assay, Qur-Chr-loaded MSNsChi NPs demonstrated superior cytotoxicity across all dosage levels in comparison to free Chr, free Qur, and free Qur-Chr nanoformulations. These findings highlight the enhanced anticancer efficiency achieved through nanocarrier-based delivery of Qur-Chr. Several findings have been conducted to validate the efficiency of NPs-based delivery systems for encapsulating cytotoxic anticancer agents across various cell lines^39,41^. In keeping with our findings, previous study has shown that encapsulating Chr, Qur, and other drugs within nanocarriers enhances their cytotoxic effects, thereby providing additional support for our conclusions^42,43^. These investigations typically focus on how such NPs modulate key oncogenic pathways, including cell death, proliferation, and cell cycle, as well as the activity of specific mutated genes implicated in carcinogenesis in diverse tumor types^44^.

Alkahtani et al. designed mesoporous SBA-15 silica nanoparticles loaded with Qur to explore their effects on proliferation and apoptosis in A549) cells. Their results presented that the Qur-loaded SBA-15 system efficiently suppressed cell growth and triggered apoptosis, emphasizing the capacity of MSN-based nanoplatforms as a potential therapeutic strategy for lung cancer^45^.

In curent work, we examined nuclear changes related with apoptosis in A549 cells, using DAPI staining to visualize chromatin structure. Importantly, quantitative assessment revealed a substantially higher proportion of apoptotic cells in cultures treated with Qur-Chr-loaded MSNsChi NPs compared to those treated with free or single-loaded drug formulations. This notable difference underscores the superior apoptotic efficacy of dual-drug-loaded NPs. These observations propose that the NPs formulation efficiently induces apoptosis at the nuclear level^46^. Remarkably, these findings are consistent with previous studies involving the loading of one or more drugs into MSNs, further corroborating the therapeutic potential of this nanocarrier-based strategy in cancer treatment^47, 48^.

Our underlying hypothesis proposed that DNA damage serve as the primary catalyst for cell cycle disrubtion. It is well established that cells with damaged DNA tend to accumulate in either the G1 or G2/M phases, while those with irreversible damage are often relegated to the sub-G1 phase^49^. Consistent with this mechanism, our results reveal that both free Qur-Chr and Qur-Chr-loaded MSNsChi NPs activate apoptosis by altering cell-cycle progression, leading to an increased population of cells in the sub-G1 phase. This findings suggests that the MSNsChi-NP formulation of Qur and Chr effectively impede proliferation in the A549 cell, offering its promise as a potential anticancer therapeutic.

Data analysis revealed a significant increase in cell death within the A549 cell population upon exposure to Qur and Chr. These findings strongly suggest a potential link between cell cycle progression and the induction of apoptosis, with enhanced endocytosis and release kinetics of encapsulated Qur-Chr formulation likely playing a key role in this process. Furthermore, accumulating evidence supports the notion that encapsulating drugs into nanocarriers can enhance apoptosis or inhibit proliferation across a range of cancer cell types. Notably, his has been demonstrated in lung cancer^50^, human ovarian carcinoma^44^, breast cancer^51^, and cervical cancer^52^ as substantiated by multiple studies. Such observations underscore the promising potential of Qur-Chr in cancer therapy, warranting further investigation and clinical exploration.

Consistent with the inhibitory effects Chr observed on the migration of human melanoma cells^53^, glioblastoma cells^54^, and triple-negative breast cancer (TNBC)^55^, our findings underscore the remarkable potential of Qur-Chr-loaded MSNsChi NPs in impeding the migration of A549 cells, as evidenced in wound healing experiments.

Extensive research has highlighted the pivotal role of Cyclin D1 in promoting cancer cell invasion and metastasis through the regulation of pathways associated with cell motility and tissue invasion^56^. As a result, Cyclin D1 has emerged as a promising therapeutic target for preventing metastasis^57^.Therefore, the observed reduction in Cyclin D1 expression in our study provides compelling evidence of its role in suppressing cancer cell migration. This mechanistic insight not only deepens our understanding of the inhibitory effects of Qur-Chr-loaded MSNsChi NPs on A549 cell migration but also presents a potential avenue for developing targeted therapies to mitigate metastatic spread in cancer.

In this comprehensive investigation, our focus extended beyond evaluating the cytotoxic, anti-proliferative, and anti-migratory effects of free Qur, free Chr, free Qur -Chr, and Qur-Chr-loaded MSNsChi NPs. We further explored into the molecular mechanisms underlying these effects by analyzing the expression patterns of key anti-apoptotic and pro-apoptotic genes in the treated cells. Previous studies show that upregulation of pro-apoptotic genes (Bax, Fas, p53) and downregulation of anti-apoptotic genes (Rbp, Cyclin D1, Bcl2) significantly reduces cancer cell survival^47,58^. Our findings are consistent with these observations, emphasizing the pivotal role of Qur-Chr in disrupting the delicate equilibrium between cell survival and apoptosis. Furthermore, our study uncovered significant alterations in the mRNA expression levels of anti-apoptotic and pro-apoptotic genes in cells treated with Qur-Chr-loaded MSNsChi NPs, in comparison to those treated with free Chr, free Qur, and free Qur-Chr. This results underscores the profound impact of encapsulating Qur-Chr within nanocarreirs, which appears to induce substantial changes in the cellular gene expression profiles. Collectively, these findings not only enhance our understanding of the molecular mechanisms underlying the therapeutic effects of Qur-Chr-loaded MSNsChi NPs but also accentuate their potential as a nanotherapeutic paltform capable of exactly modulating apoptotic pathways cancer cells.

While the results are encouraging, there are some important limitations to keep in mind. All experiments were done in vitro, which doesn’t fully capture the complexity of a tumor in a living organism. Features like tumor heterogeneity, interactions with the immune system, and how NPs are cleared from the body could all effect the treatment’s effectiveness and safety. Because of this, translating these results to clinical settings will need careful assessment and further study. Furthermore, this study did not examine long-term toxicity or potential off-target effects, so these issues will need to be methodically addressed in future studies.

## Conclusion

In this study, we developed a drug delivery system based on MSNs-Chi NPs loaded with Chr and Qur for the efficient delivery of therapeutic agents to A549 lung cancer cells. Qur MSNsChi NPs, Chr-MSNsChi NPs and Chr-Qur@MSNsChi NPs were successfully synthesized, and the their formation was confirmed by FTIR, XRD and SEM. The MSNsChi NPs exhibited high encapsulation efficiency and sustained drug release profiles. Cytotoxicity assays performed on A549 cancer cells demonstrated significantly lower IC_50_ values for Chr- Qur @MSNs-Chi NPs compared with the free drugs formulations, indicating enhanced therapeutic efficacy. Encapsulation within MSNs-Chi NPs enabled prolonged release of Chr-Qur combination, thereby reducing the effective dosage required for killing cancer cells. Additionally, these NPs facilitated efficient intracellular delivery of Chr-Qur, leading to increase dapoptosis and a pronounced reduction in cell viability, proliferation, and migration of cancer cells compared to the free compounds. This approach holds promise for reducing drug resistance and adverse effects in lung cancer treatment. Moreover, the dual-drug co-loading strategy in MSNs-Chi NPs effectively addresses solubility, stability, and metabolic degradation challenges, providing a robust and synergistic nanocarrier platform superior to conventional liposomal or polymeric systems. For future research, we suggest performing in vivo studies to validate these findings in animal models. It will also be significant to carry out pharmacokinetic and biodistribution analyses to measure systemic exposure, clearance, and target specificity.

## Data Availability

All data generated or analyzed during this study are included in this published article.
